# Asthma and the Missing Heritability Problem: Necessity for Multiomics Approaches in Determining Accurate Risk Profiles

**DOI:** 10.3389/fimmu.2022.822324

**Published:** 2022-05-25

**Authors:** Tracy Augustine, Mohammad Ameen Al-Aghbar, Moza Al-Kowari, Meritxell Espino-Guarch, Nicholas van Panhuys

**Affiliations:** Laboratory of Immunoregulation, Systems Biology and Immunology Department, Sidra Medicine, Doha, Qatar

**Keywords:** asthma, GWAS - genome-wide association study, inheritability, epigenetics, microbiome and dysbiosis, maternal inheritance, atopic disease, multiomics approach

## Abstract

Asthma is ranked among the most common chronic conditions and has become a significant public health issue due to the recent and rapid increase in its prevalence. Investigations into the underlying genetic factors predict a heritable component for its incidence, estimated between 35% and 90% of causation. Despite the application of large-scale genome-wide association studies (GWAS) and admixture mapping approaches, the proportion of variants identified accounts for less than 15% of the observed heritability of the disease. The discrepancy between the predicted heritable component of disease and the proportion of heritability mapped to the currently identified susceptibility loci has been termed the ‘missing heritability problem.’ Here, we examine recent studies involving both the analysis of genetically encoded features that contribute to asthma and also the role of non-encoded heritable characteristics, including epigenetic, environmental, and developmental aspects of disease. The importance of vertical maternal microbiome transfer and the influence of maternal immune factors on fetal conditioning in the inheritance of disease are also discussed. In order to highlight the broad array of biological inputs that contribute to the sum of heritable risk factors associated with allergic disease incidence that, together, contribute to the induction of a pro-atopic state. Currently, there is a need to develop in-depth models of asthma risk factors to overcome the limitations encountered in the interpretation of GWAS results in isolation, which have resulted in the missing heritability problem. Hence, multiomics analyses need to be established considering genetic, epigenetic, and functional data to create a true systems biology-based approach for analyzing the regulatory pathways that underlie the inheritance of asthma and to develop accurate risk profiles for disease.

## Introduction

To address the epidemic of asthma currently faced by society, it is necessary to gain a deeper understanding of the interwoven elements that lead to the initiation of the disease state. Why a significant proportion of the population suffers from asthma, while the rest remain healthy, has yet to be adequately determined. Asthma is a serious worldwide health issue and is now counted among the most common of chronic conditions in industrialized nations with over 300 million people affected worldwide and approximately 250,000 deaths recorded on a yearly basis (1). While the initial sensitization to environmental allergens that is responsible for the occurrence of atopic asthma typically occurs during childhood, we postulate that the predisposition to develop atopy occurs very early in life. Mediated through the inheritance of genetic risk factors which act in combination with multiple non-genetically encoded factors that are passed on either prior to birth or in the immediate post-natal period leading to the establishment of a predisposition for atopic sensitization (2) and the development of asthma.

The incidence of asthma has rapidly increased by 3-5 fold over the last 40 years (3, 4). During this time, a large number of genetic contributors to asthma have been identified (5), indicating a significant genetic component to the disease. However, the rapid rise in the rate of disease, coupled with the strong familial association, indicates that this dramatic increase is also the result of alterations in non-genetically encoded heritable factors (6, 7). As such, asthma can be characterized as a highly complex syndrome with genetic, epigenetic, and environmental risk factors that contribute to the establishment of the disease. Multiple genetic components have been identified as either protective or contributory to disease susceptibility; with interactions between genetic, epigenetic, and non-genetic factors then setting a threshold of susceptibility for an individual’s environmental exposure to sensitizing allergens governing the onset of disease. Additionally, alterations in lifestyle associated with modern industrialized living conditions may be responsible for the increased risk, ultimately leading to the induction of disease in a growing proportion of the population (8). Interestingly, the major risk factor for developing asthma has been shown to be a familial history of disease (9), with maternal rates of inheritance contributing significantly more than paternal transmission rates (5-fold greater) (10). Estimations from GWAS indicate that the single nucleotide polymorphisms (SNPs) currently identified are only able to account for 2.5%-14% of asthma heritability (5). Whereas twin studies have shown asthma heritability rates in the adult population of up to 55%, with even higher estimates reported among young children (11, 12). The low rates of currently identified variants accounting for the heritability of asthma points to the importance of additional non-genetically encoded factors playing a role in the susceptibility to asthma and atopic sensitization. These observations have led to the development of ‘the missing heritability problem’, as a theory to help explain the limitation of GWAS and other genetic studies to elucidate the variance in heritability of disease (13).

## Missing Heritability Theory

The advent of GWAS techniques along with the ability to sequence and identify variants that contribute to complex human diseases and traits has allowed the study of inherited predisposing factors, far beyond the scope provided through family linkage studies; with the latter previously proving to be powerful in the identification of Mendelian disorders caused by single gene variants (14).

Next generation sequencing (NGS) has driven the development of new tools for assaying the relative impact of rare variants that have much more modest effects in contributing to the phenotypic outcome (13). Inclusion of these rare variants into multiple variant analysis to account for the development of specific conditions has proved to be highly informative, where the variants have a relatively large effect on disease development (>two-fold increase).

**Figure 1:**
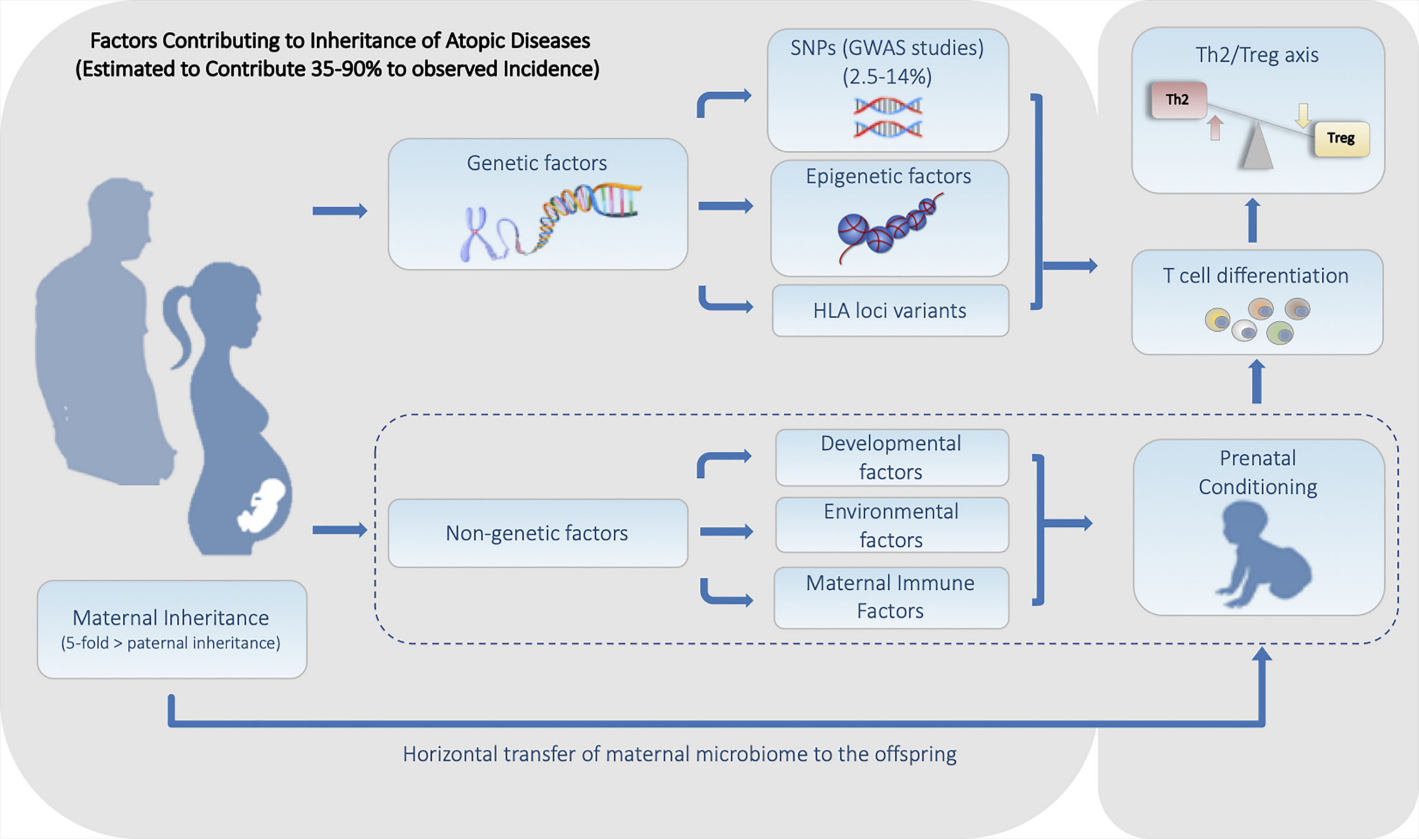
Summary of the Heritable Genetic and Non-Genetic Factors that Contribute to the Predisposition for Atopic Diseases. Factors can be divided into two major categories. (1) Genetic factors including genetically encoded features such as SNPs and HLA haplotype, along with alterable non-genetically encoded epigenetic features including DNA methylation and histone acetylation status. (2) Non-Genetic factors consisting of developmental, environmental, and maternal influences including the maternal microbiome is all transmitted during fetal and/or early neonatal development.

In the classical example of macular degeneration (15), five discrete loci account for 50% of the genetic heritability observed. Conversely, early studies into the variants responsible for human height could only account for approximately 5% of the genetic heritability observed through twin studies (estimated at 50%-80%), with over 50 variants associated with human height (16). Whereas, more recent studies have been able to account for up to 45% of the genetic association between SNPs and height, by adapting the analysis of individual SNPs that contribute to the variance of a characteristic through the use of models that are able to simultaneously consider multiple SNP associations (16). These early studies identified a still unresolved problem in using GWAS to analyze inherited characteristics now known as the ‘missing heritability problem’ (13). Part of the issue in using such analysis lies in the fact that many of the identified SNPs have been shown to only confer a minor increase in the disease odds ratio (1.1-1.4), combined with the trend for these variants to be present in the population at low to rare minor allele frequencies. Asthma, similar to other complex traits, likely has significant degrees of heritability stemming from multiple non-genetically encoded factors, which may account for much of the ‘missing heritability problem’ observed when trying to calculate risk scores from GWAS results. This is evident when considering both the degree of heritability and the dramatic rise in the rate of asthma incidence in developed countries that have been observed over the past 40 years. Together, these data indicate that a significant interplay exists between the inheritance of predisposing genetic factors, with the inheritance of more malleable and adaptive components, including those epigenetic factors that work in concert with developmental and environmental cues (**Figure 1**). All of which need to be considered in order to fully understand the heritable risk factors associated with asthma inheritance (17).

## GWAS Discovery of Asthma Associated Genetic Linkages

The first GWAS of asthma was published in 2007 by Moffatt et al. who focused on mapping the effects of SNPs associated with childhood asthma and revealed the association of the *ORMDL3* gene with susceptibility to asthma (18). Over the past 15 years, GWAS have identified tens of thousands of additional SNP variants associated with asthma-susceptibility loci mapped to both coding and non-coding regions of the genome (5, 19, 20). In a previous review by Kim et al. (20), the authors summarized the results from GWAS of asthma from the years 2008 to 2018 to show the importance of merging the GWAS findings with deep learning approaches to understand the genetic architecture of the asthma. To summarize the up-to-date results of GWAS of asthma, here we searched for “asthma” using the GWAS catalog database. A total of 29 asthma GWAS studies were conducted from July 2018 to November 2021 and an estimated 2000 SNPs were identified with 580 SNPs being replicated in at least two GWAS studies. (see **Table 1**: Summary of replicated SNPs in more than 4 GWAS.)

**Table 1:** Asthma Associated SNPs replicated in GWAS from July 2018 to November 2021.

SNP ID	Region	Mapped genes	Ref Allele	Alt Allele	GWAS replication studies								
**rs1837253**	5q22.1	*BCLAF1P1, TSLP*	T	C	(21)	(22)	(23)	(24)	(25)	(19)	(26)	(27)	(28)
**rs34290285**	2q37.3	*D2HGDH*	G	A	(21)	(22)	(28)	(27)	(23)	(24)	(25)	(19)	
**rs117710327**	19q13.11	*SLC7A10, CEBPA*	C	A	(21)	(22)	(28)	(29)	(27)	(25)	(19)		
**rs72743461**	15q22.33	*SMAD3*	C	A/T	(21)	(22)	(23)	(28)	(25)	(27)			
**rs11071559**	15q22.2	*RORA*	C	T	(21)	(24)	(25)	(19)	(28)	(29)	(27)		
**rs12365699**	11q23.3	*Y_RNA, CXCR5*	G	A	(21)	(22)	(24)	(28)	(25)	(19)			
**rs3122929**	12q13.3	*STAT6*	C	T	(21)	(28)	(22)	(30)	(24)				
**rs72823641**	2q12.1	*IL18R1, IL1RL1*	T	A/C	(21)	(22)	(28)	(29)	(24)	(25)			
**rs12413578**	10p14	*LINC02676, LINC00709*	C	G/T	(21)	(28)	(22)	(25)					
**rs12964116**	18q21.33	*SERPINB7*	A	G	(21)	(22)	(24)	(28)	(19)				
**rs16903574**	5p15.2	*OTULINL*	C	A/G	(21)	(22)	(24)	(28)	(25)				
**rs479844**	11q13.1	*AP5B1, OVOL1*	A	G	(21)	(28)	(24)	(28)	(19)	(29)			
**rs5743618**	4p14	*TLR1*	C	A/G	(21)	(29)	(27)	(24)	(25)	(19)			
**rs61816761**	1q21.3	*FLG, FLG-AS1*	G	A/T	(22)	(23)	(24)	(25)	(19)				
**rs7936312**	11q13.5	*LINC02757, EMSY*	G	T	(21)	(22)	(23)	(24)	(28)				
**rs992969**	9p24.1	*GTF3AP1, IL33*	A	C/G/T	(21)	(22)	(24)	(25)	(19)	(27)			
**rs12722502**	10p15.1	*IL2RA*	C	T	(21)	(22)	(25)	(19)					
**rs13099273**	3q28	*LPP*	A	G/T	(22)	(25)	(19)	(29)					
**rs2070901**	1q23.3	*FCER1G*	G	C/T	(21)	(22)	(28)	(19)	(27)				
**rs3024971**	12q13.3	STAT6	T	G	(22)	(28)	(29)	(19)					
**rs35570272**	3p22.3	*GLB1*	G	T	(21)	(24)	(28)	(25)	(19)	(29)			

Several of the largest studies have been performed in the UK using data from the Trans-National Asthma Genetic Consortium (23,000 asthma cases versus 118,000 controls) (31) and the UK Biobank (64,000 cases versus 329,000 controls) (28). A meta-analysis of these two studies revealed 167 significantly associated loci of which 66 were novel loci; further accounting for an additional 1.5% of the reported asthma heritability, bringing the total estimate of heritability to approximately 8-9% (19). Together, these findings underscore the difficulty of determining the genetic basis of complex disorders, even when using extremely large cohorts and indicate that non-genetically encoded inherited features may be key to developing a comprehensive understanding of the inherited incidence of asthma.

A large percentage of the SNPs identified *via* GWAS are non-coding variants, mapping to either intergenic or intronic regions and are currently lacking biological evidence for their functional relevance (5). Studies of GWAS data combined with functional annotations and quantitative trait loci (QTL) have sought to strengthen their context-specific relevance. In addition to replicating previously known loci, new candidate genes including *DGKQ, SLC26A1*, and *IDUA* genes, as well as variants of genes encoding transcription factors like *NF-KB*, *cJUN*, STAT family, *ERG1*, *ELF1*, *EBF1*, and the GATA family have been identified (32–35). Atopy-associated GWAS have identified multiple relevant biological pathways, including autoimmune and inflammatory response pathways, thereby providing insights into the underlying heritable risk (31, 36). Interestingly, asthma-risk variants identified in several studies have been mapped to 17q12-21 locus, including *ORMDL3, GSDML*, and *CDHR3* genes (35, 37–41) and have yielded several functional validations (42–44). In addition, variants of the *TSLP* gene, a cytokine secreted by epithelial cells that initiates allergic inflammation (45) have been replicated in several studies suggesting that this could be a potentially critical target for drug development (5, 35, 46, 47).

**Figure 2:**
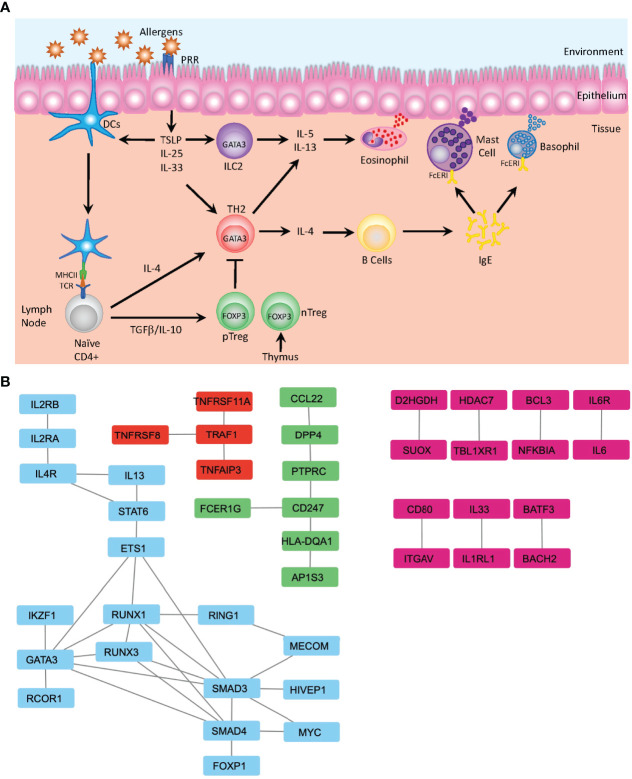
Mechanisms of Allergic Disease Development. **(A)** Representative image depicting the first stage of an allergic response driven by the recognition of environmental allergens as inflammatory mediators by pattern recognition receptors (PRR) present on the surface of dendritic cells (DC) and epithelial cells in barrier tissues. Epithelial cells produce defensive cytokines including TSLP, IL-25 and IL-33 that modulate activated DC and direct naïve T cells towards the TH2 phenotype. In the absence of inflammatory stimuli, naïve T cells may be activated in a tolerogenic manner and differentiate to become peripherally induced Treg cells (pTreg), which in concert with thymically produced T regs (nTreg), mediate the dampening of inflammatory responses. Activated TH2 cells produce IL-4 and mediate B cell class switching and production of IgE. Together with ILC2, Th2 cells additionally produce IL-5 and IL-13 that regulate the activation of the innate immune system and induces migration of eosinophils, mast cells and basophils to the sites of inflammation, where IgE bound to the FcERI receptors on mast cells and basophils recognizes the cognate allergen. This induces degranulation and release of further inflammatory mediators including histamine, leukotrienes, and heparin. **(B)** Analysis of protein-protein interactions of the 216 genes identified by GWAS from Han et al. (19) using the STRING database (48). Identification of several clusters of highly relevant pathways that overlay with the known functional pathways previously associated with asthma and allergic diseases, including the major cytokines signals associated with TH2 differentiation (IL-2, IL-4 and IL-13) along with the downstream mediators of differentiation—STAT6 and GATA3—and transcription factors associated with Treg differentiation—FOXP1 and SMAD—which are involved in the TGFβ signaling (blue). Additionally, the TRAF1 signaling nexus is identified, where TRAF1 has previously been identified as a key factor in allergic inflammation and the regulation of IL-4 production (red) (49). FcERI signaling and interactions between TCR and MHCII (HLA-DQA1) are identified (green) and multiple pairs of protein-protein interactions integral for development of allergic inflammation are also identified (pink)—notably IL-33-IL1RL1 (50) and BATF3-BACH2 (51). Data displayed is developed using Cytoscape v.3.9 (52).

Many of the identified SNPs are known to be associated with pathways involved in the development and differentiation that regulate either the induction of inflammatory Th2 cells, which promotes the onset and progression of atopic asthma or are associated with maintenance of the Treg subset that is required to mediate immune homeostasis and tolerance to both self and innocuous environmental antigens (**Figure 2A**). Th2-associated genes identified include the master regulator of differentiation *GATA3* and several genes associated with its upstream signaling pathways, including *STAT6, IL4R, TSLP*, and *IL-33R*. Further, multiple genes associated with Treg differentiation have also been implicated, including *FOXP1* (53) and *FOXP3* (54). Defects in *FOXP3* can lead to an inability to generate Treg cells (55), leading to the spontaneous induction of allergic disease, further demonstrating the essential role of Treg cells in controlling spontaneous reactions to common environmental stimuli and the suppression of Th2 mediated diseases (56). GWAS have also detected multiple variants affecting Treg-related differentiation-promoting genes that are also associated with an increased risk of asthma, such as *BACH2, IL2Rb*, and *SMAD3* (5) (**Figure 2B**).

It is important to note that genome-wide findings have some limitations in terms of the quality and quantity of samples gathered and methodological biases which may lead to significant false-positive associations. Additionally, the highly heterogeneous nature and multiple endotypes of complex diseases such as asthma (57) need to be adequately controlled. The lack of adequate controls potentially lead to studies being conducted that have been hampered by poor design, such as a lack of specificity in disease characteristics being used in order to gain a sufficiently large population size. Also, ignoring the effects of environmental interactions on disease subtypes potentially could lower the number of asthma-risk variants identified to date (17). Multiple asthma endotypes have now become well recognized, with atopic asthma being the most common form, affecting from 70%–90% of childhood and approximately 50% of adult patients (1). Patients with this phenotype typically have high levels of Th2 cytokines, significant eosinophilia, and high FeNO. In addition to atopic asthma, several other endotypes can be confirmed by differential diagnosis including: allergic bronchopulmonary aspergillosis, allergic bronchopulmonary mycosis, aspirin-exacerbated respiratory disease, late onset eosinophilic, paucigranulocytic, neutrophilic, and smoking related asthma (57, 58). In addition to recruitment of asthmatics for GWAS studies being complicated with multiple distinct endotypes and phenotypes, studies into rates of asthma misdiagnosis consistently report rates as high as 30% (59, 60). As the minimum criteria for inclusion in asthma related GWAS may only require the self-reporting of asthma or a previous medical diagnosis of asthma, this infers that a non-significant proportion of asthmatics included in large scale GWAS may not actually suffer from the disease, further confounding outcomes. On the contrary, a classification of asthma based on distinct phenotypes and endotypes allows for a more accurate clinical definition of the exact nature of the inflammatory response underlying disease and ultimately allows for the design of an optimal recruitment strategy. Where the phenotype reflects the observable characteristics of disease in a patient and endotype refers to the underlying biological processes that give rise to the observed properties of the phenotype (61). As such, specific biomarkers for disease endotypes as measurable characteristics of asthma subtype are becoming increasingly more essential for clinicians and researchers to complement a clinical diagnosis of phenotype and to identify the specific phenotypes and endotypes of asthma. Current biomarkers for endotype include such factors as IgE, allergen specific IgE (sIgE), eosinophil and neutrophil counts in blood and sputum, fraction of exhaled nitric oxide (FeNO), urinary Leukotriene E4, and an increasing array of SNPs (57). This approach is already suggesting entirely novel pathways to disease—e.g., alternative macrophage specification, steroid refractory innate immunity, the interleukin-17–regulatory T-cell axis, epidermal growth factor receptor co-amplification, and Th2-mimicking but non-T-cell, interleukins 18 and 33 dependent processes that can offer unexpected therapeutic opportunities for specific patient endotypes (62).

The use of GWAS analysis in combination with other genetic analysis tools, including expression quantitative trait loci (eQTL), admixture mapping, and polygenic risk scores (PRS), in addition to the study of the underlying biological pathways identified, may help to better understand the etiology of the disease. eQTL analysis is an effective tool used to identify the effects of genetic variations on gene expression analysis through direct testing of association between genetic variations and gene expression levels. In a recent eQTL analysis study, the authors generated a list of 154 asthma-associated genes and observed that the top 30 polymorphisms were linked to multiple gene transcripts, indicating that a single SNP may be responsible for regulating the function of multiple genes (63). Conversely, eQTL analysis has also been used to show a strong relationship between *ORMDL3* transcription levels and multiple asthma-associated SNPs (18). Conversely, admixture mapping is a tool used to study genetic landscapes and assess the differential risks associated with the disease, allowing for the ancestry specific genomic risk association of a trait identified by GWAS to be compared against other ancestries and to look for alterations in the odds ratio associated with inheritance. The prevalence of asthma in African American and Latino/Hispanic populations are notable examples of where admixture mapping has yielded abundant data. Studies have identified genes responsible for both increases in the prevalence of asthma and a reduction in the response to common asthma medications in African American admixture populations when compared to those with European American ancestry (64). The genetic contribution to complex traits such as asthma, likely results from the accumulation of multiple SNPs with relatively minor effects. As such, the calculation of PRS, which has previously been widely used in animal and plant breeding (65) is a relatively new approach to better understand an individual’s overall inherited risk of disease. PRS values are obtained by weighting the effect size of the risk allele from the GWAS to the sum of the genomic risk alleles of an individual. Calculation of PRS is being rapidly applied in the public health sector to accelerate personalized preventive, diagnostic, and therapeutic strategies (66–68). A recent Canadian study, utilizing both GWAS loci previously identified in the literature and those found in two Canadian cohorts, explained that 37% of the variance in disease incidence was seen in a Canadian population (69). Allowing PRS to then be used to identify individuals who may benefit the most from timely interventions, such as the use of emollient moisturizers (70) or early introduction of potential food allergens as seen in the LEAP and EAT studies (71, 72). It was shown that the introduction of common allergens including peanuts (LEAP) and milk, peanut, sesame, fish, egg, and wheat (EAT) during the first year of life could prevent allergy in infants identified as either high-risk, sensitized, or non-sensitized. Despite these significant advances in identifying the underlying causes of allergic disease provided through GWAS and other forms of genetic analysis, there are still significant gaps in our current models of the disease development that can likely be filled through analysis of other, non-genetically-encoded forms of inheritance.

## Epigenetic Inheritance of Asthma

Relatively recently, the field of asthma epigenetics has been greatly advanced with the introduction of epigenome-wide association studies (EWAS), in which changes in DNA methylation across the whole epigenome, of either particular cells or tissues, can be investigated to draw associations to asthma incidence. An excellent example of what can be accomplished using EWAS type analysis was recently provided following the CpG methylation meta-analysis from the Pregnancy and Childhood Epigenetics (PACE) consortium, in which over 3,000 sites were identified in newborns as being associated with maternal smoking, including several linked to an enhanced risk of asthma (73). Additionally, the application of EWAS has helped in the identification of differentially methylated regions in asthma-related genes including *IL-4, IL-13,* and *RUNX3* (74). EWAS was also able to detect hypomethylation in genes related to eosinophils and cytotoxic T cell activation in whole blood of childhood asthmatic patients (75) and EWAS of sputum samples revealed the hypermethylation of the PCDH2 gene. This is an important factor in the maintenance of airway epithelial connectivity which has also been associated with asthma (76) and may play an integral role in barrier defense, similar to filaggrin in atopic dermatitis (77).

Further, a distinct CpG methylation profile associated with asthma was also found to be present following an EWAS meta-analysis of the cord blood collected from 8 cohorts of newborns (78). EWAS meta-analysis has also been applied to investigate cytokine interaction pathways related to asthma in blood of children with atopic asthma, identifying 35 hypermethylated and 95 hypomethylated genes associated to asthma development (79). In adults, DNA methylation associated with asthma has been applied to PBMCs, including eosinophils, neutrophils, and monocytes identifying 9 common hypermethylated genes. It also highlighted common pathways and gene expression networks associated with asthma, including 3 pathways in eosinophilic asthma and a novel network related to the Wnt signaling pathway in neutrophilic asthma (80). Moreover, several differentially methylated genes associated with asthma have been identified from nasal brushings of childhood atopic asthma patients (nasal methylome) (81), with several studies indicating that asthma associated epigenomic profiles obtained by EWAS can be tissue-specific (82).

With the emergence of NGS technologies, it is increasingly easier to study the developmental origins of asthma and to assess the epigenetic factors that contribute to the induction of disease (83). In particular, it is now possible to assess how fetal conditioning plays a role in setting thresholds for disease development by controlling genetic-epigenetic-environmental interactions. Previously, traditional immunological studies, GWAS, and studies of monogenic diseases with associated atopy have determined that CD4+ T cells are a crucial component in the establishment of asthma and other atopic diseases (84). Studies on the epigenetic regulation in these populations have revealed that both Th2 and Treg cell populations can be influenced in a heritable manner, with epigenetic changes at the *IL4* promoter being associated with a predisposition to develop asthma (85, 86). In Tregs, the permissiveness of histone methylation at conserved non-coding sequences and the relative abundance of histone acetylases in comparison to histone deacetylases have emerged as key epigenetic factors linking Treg development with disease susceptibility (87). Additionally, alterations in the DNA methylation patterns in DC2 dendritic cells that prime Th2 differentiation have been observed (88). Human birth cohort studies have shown distinct methylation signatures of *IL-4R* and *GATA3* genes being present at birth in children who developed asthma (89). Methylation of *SMAD3* at birth, a locus previously identified by GWAS and known to regulate *FOXP3* induction during Treg development (90) was also found to be strongly associated with the development of childhood asthma, potentially through a dysregulation of IL-1b signaling (91). Studies in both animal models and humans (85, 86) have shown evidence for the epigenetic heritability of allergic disease across generations (88). This leads to the theory that disease incidence can be passed to the next generation, both by shared environmental factors and by “induced epigenetic transmission” through both histone modifications and DNA methylation patterns, *via* the formation of metastable epialleles (92). Additionally, evidence of epigenetic transmission has been gained from studies on the impact of maternal smoking during pregnancy that showed increases for the risk of asthma in children (93, 94). Also, induction of differential DNA methylation in genes involved in the fundamental developmental processes of offspring of mothers who smoked (95) and, as mentioned above, a meta-analysis of 13 cohorts by the PACE consortium which identified nearly 3,000 CpG sites corresponding to differentially methylated genes in response to maternal smoking, including the E*SR-1, ASRT* and *IL-32* genes were previously implicated in genetic studies of asthma (96). Furthermore, exposure to diesel particulate matter in mice during pregnancy was found not only to increase the incidence of asthma sensitivity in the F1 generation but also increase the sensitivity in the F2 and F3 generations. This finding correlates distinct and persistent changes in the methylation status of multiple genes with various chromatin modification pathways (97) and raises the possibility of the enhanced risk of asthma being transferred epigenetically through multiple generations *via* trans-generational epigenetic inheritance (98).

Together, these studies highlight the profound effects of epigenetic landscape alterations on the inheritance of asthma and the need to include an analysis of the impact of differential epigenetic modifications on gene expression when assessing potential asthma risk factors.

## Asthma and the Transfer of Maternal Immune Factors

Previous studies report that maternal asthma is a greater risk factor than paternal asthma for the inheritance of the disease (5- fold greater) (9) and a lack of maternal asthma control during pregnancy imparts a greater risk of asthma/recurrent wheeze in the offspring when compared to a controlled maternal asthma status throughout pregnancy (9). During *in utero* development, the fetus is constantly exposed to an environment in which it is surrounded by the maternal metabolic and immune milieu (99). A growing number of studies indicate that the *in utero* environment of asthmatic mothers is distinct from that of non-asthmatic mothers, where the presence of those cytokines associated with asthma may cause developmental alterations (100), with reports indicating that both asthma and atopy have their origins during *in utero* development (93, 100). Maternal atopy has been found to be associated with differential methylation patterns in the cord blood of neonates (99). A study of 36 patients enrolled in the Infant Immune Study looking for DNA methylation signatures predictive of childhood asthma identified that cord blood mononuclear cells (CBMCs) harbored 589 differentially methylated regions (DMRs) associated with childhood asthma; further analysis of which showed a significant enrichment of DMRs in genes controlled by immunoregulatory *TGF-β1* and *pro-inflammatory IL-1β* (101). Moreover, CBMCs of asthmatic children from asthmatic mothers secreted more of IL-1β and exhibited high SMAD3 methylation as compared to non-asthmatic children from asthmatic mothers (101). Additional cord blood studies show that the association of increased methylation of a functional CpG site in the IL-2 promoter increased the likelihood of severe asthma exacerbations, as well as hospital admissions for asthma/wheeze in children between 2 and 8 years of age (102). Moreover, Barton et al. showed that higher methylation of GATA3 CpGs-2211/-2209 at birth was associated with a reduced risk of asthma at 3 years of age (103). A very recent EWAS of cord-blood samples in a cohort of 96 mother-child pairs, recruited as part of the ongoing ELMA- Epigenetic Hallmark of Maternal Atopy and Diet study, indicated that maternal atopy was associated with specific epigenetic signatures in the offspring, identifying 83 CpG islands mapping to 50 genes associated with maternal atopy (99). The genes *C20orf166, STAC, SYT8, KCNJ15, SCD, LINCOO669, PLEKHA2, ITM2C, NT5C3A* and *NPEPL1* had the most significant differentially methylated sites of which *SCD, ITM2C, NT5C3A* and *NPEPL1* genes are known to impact the immune system, allergy, and asthma; thereby suggesting that maternal atopy constitutes a unique intrauterine environment which is associated with the induction of distinct methylation patterns associated with the inheritance of a pro-atopic state (99).

There is also evidence to suggest that the transmission of maternally derived immune factors during fetal development may be a significant source of inherited immunological conditioning. As *in utero* exposure to stress response factors (104) and inflammatory cytokines (105, 106) have both been found to be important risk factors for the development of asthma. During development, fetal and neonatal immune responses are considered immature and present significant differences to those in adults, displaying a significant skewing towards the development of type 2 inflammatory responses, as part of a mechanism that protects against fetal rejection by the maternal immune system (107). Maternal cytokine production levels have been shown to influence the development of the immune system, with higher maternal IFN-γ:IL-13 and IFN-γ:IL-4 ratios during pregnancy being associated with decreased risk for childhood asthma at the age of 5 (100) and a decreased IFN-γ:IL-4 ratio during the first trimester of pregnancy being associated with an increased risk of atopy (108). This coincides with an increased rate of Th2 cells and a significant decrease in Treg: Th2 cells ratio present at birth in the cord blood of children born to atopic mothers (109). Together, these data suggest that the bias towards generating a type 2 inflammatory response during fetal development may carry over into neonatal life, where exposure to otherwise innocuous environmental antigens could induce an inflammatory response, as opposed to a tolerogenic response.

Among other maternal factors thought to play a potentially significant role in the development of asthma are the presence of maternal cells in fetal lymph nodes, which occurs through a process known as maternal microchimerism (MMc) (110). While the presence of MMc has been found to be associated with chronic inflammation and autoimmune diseases, it has also been shown to be protective against the development of asthma (111). The transplacental migration of maternal cells leads to their localization in fetal lymph nodes, where fetal CD4+ Tregs have been shown to preferentially develop following exposure to maternally associated non-inherited antigens, creating an early tolerogenic environment (112). These initial fetal clonotypes have then been observed to exert a lasting influence on the development of the T cell repertoire which can be observed in the persistence of clonotypes into adulthood (113).

Breastfeeding provides a further potential avenue for the vertical transmission of familial traits. As the complement of maternal immunoglobulins, cytokines, and immune cells contained in breastmilk provide an early form of defense during the initial conditioning of the neonate to the external environment (114) and may play a key role in setting immune tolerance thresholds to potential allergens; an aspect of which is especially important during the first few weeks of life when the infant gut remains highly permeable to both macromolecules and cellular components (115). Currently, no conclusive mechanistic evidence exists for how the transfer of maternal factors is protective against asthma, but multiple meta-analyses have shown protective effects of breast feeding against the induction of asthma and atopic disease [reviewed extensively in (116)]. However, a recent study by Ramanan et al. (117) demonstrated that the transmission of maternal Ig *via* breast milk was able to induce a multigenerational setpoint that governs the generation of gut resident Tregs responsible for induction of tolerance. Establishment of a protective population of RORg+ Treg was shown to be dependent on the maternal transmission of IgA and was reliant on a double negative feedback pathway whereby RORg+ Treg and IgA+ B cells co-regulate each other. Transmission of maternal IgA depressed the induction of RORg+ Tregs, allowing the production of commensal-specific B cell production of IgA, which could in turn be passed on to the next generation. Notably, these effects were found to be non-genetic, non-epigenetic, and non-microbial and were carried over multiple generations, even when animals were continuously backcrossed into their original genetic strain. In human subjects, patients with food allergies have been shown to have reduced numbers of RORg+ Tregs, and tolerance induction mediated by commensal bacteria has been shown to be dependent on RORg+ Tregs (118). Together, these data, built on previous findings, indicate dysbiosis in IgA recognition patterns in children with allergies who are readily observable at one month of age, a time when the IgA antibodies present are predominantly of maternal origin in breast-fed children (119). The establishment of RORg+ Treg levels by maternal Ig-based factors could then provide an initial setpoint in determining the baseline atopic state for an individual, leading to a predisposition towards food allergies and then progressing through the atopic march and asthma in later life (120, 121).

## Environmental and Microbial Factors in the Development of Atopic Disease

Apart from genetic susceptibilities, the incidence and progression of asthma are greatly influenced by early environmental exposures, with the likelihood of asthma development beginning with *in utero* exposures (93). Factors including maternal diet, environmental pollutants, maternal microbiome, and maternal consumption of antibiotics during pregnancy have all been identified as contributing risk factors (122). Maternal dietary patterns significantly influence childhood immune development and function and a balanced healthy diet during pregnancy has been shown to confer protection against childhood asthma (93). Specifically, the regular dietary intake of foods high in antioxidants, vitamin D, and omega-3 polyunsaturated fatty acids during pregnancy has been shown to have protective effects against the development of asthma and other atopic diseases (123, 124). A meta-analysis of epidemiological studies based on the maternal dietary profile during the gestational period and documentation of asthma in their offspring revealed that an appropriate dietary intake of vitamin D, vitamin E, and zinc reduces the risk of childhood wheezing (125). Additionally, a birth cohort study of mother-child pairs revealed that high maternal dietary intake of potential food allergens, including peanut, milk, and wheat, in early pregnancy could reduce the risk of mid-childhood allergy and asthma (126).

Considerable effects of early life environmental exposures have been found to play a critical role in governing the rates of childhood asthma. Multiple studies, including the PARSIFAL and GABRIEL cohorts, have shown that children born in rural farming families are more resistant to atopic diseases, including asthma, than children raised in more urban environments. This is potentially due to an exposure, during early life, to a wider range of microbes present in farming environments (127–132). Additionally, maternal exposure to a microbial environment associated with livestock has been shown to be important in shaping fetal immune responses (132). A recent study found that chronic exposure to either low-dose bacterial endotoxin (lipopolysaccharide) or farm dust conferred protection against house dust mite (HDM)-induced asthma (129). The study also revealed that levels of a ubiquitin-modifying enzyme, A20 (TNFAIP3 protein), present in the lung epithelium, were responsible for providing protection against asthma in children growing up on dairy farms. Consistent with these findings, a loss-of-function SNP present in the *TNFAIP3* gene confers an increased risk of asthma in these children (129), highlighting the strong role of gene-environment interplay in the development of asthma.

A study of mothers living in farming environments that raise livestock showed increased rates of consumption of microbial-rich unpasteurized milk, which is associated with diverse maternal gut microbiomes and, in turn, confer protective effects against asthma development in their children (93). The maternal gut microbiome composition and the microbial metabolites produced, including short-chain fatty acids (SCFAs), have a direct impact on the development of the fetal immune system (133). The effects of SCFAs on atopy were further characterized in pregnant mice fed either on a high-fiber diet or given SCFAs in drinking water, both of which conferred protection against asthma in offspring, mediated *via* the induction of enhanced Treg cell numbers and function (130). Murine studies on the transmission of mammalian gut microbiota have also shown that vertical inheritance from mother to offspring is predominant over the horizontal transmission of microbiota, although this is most likely associated with pathogenicity (128). A paired longitudinal metagenomic study of mother and child stool samples in a Finnish birth cohort identified that although the mother’s dominant gut bacterial strains were often vertically transmitted to the offspring, non-dominant strain transmissions also occurred in the offspring (131). The maternal gut microbiome has been shown to be vertically inherited from mother to child both prenatally and based on the mode of delivery (127, 131), as well as postnatally through breastfeeding (134) where the infant gut microbiome co-develops along with the intestinal immune system following birth. Vaginally delivered babies develop gut microbial communities similar to maternal vaginal microbial composition, whereas, in case of caesarean sections, babies acquire gut microbial communities similar to the maternal skin microbiome profile (135). Inevitably, the composition of the maternal microbiome during pregnancy has a significant impact on fetal immune development and influences the predisposition of the offspring to develop asthma (133). Furthermore, exposure of the fetus to probiotic bacterial species *Lactobacillus* and *Bifidobacteria* in the pregnant mother confers increased resistance to atopic diseases in these children, and this was shown to be achieved through enhanced recruitment of Tregs to the fetus through cord blood (136, 137).

Conversely, maternal exposure to antibiotics during pregnancy can increase the risk of asthma in children (138). Maternal use of antibiotics induces perturbations in the composition of the indigenous gut microbiome, resulting in microbial dysbiosis. Maternal microbial dysbiosis can, in turn, negatively affect the inheritance of a healthy maternal microbiome in the fetus. Human cohort studies have shown that prenatal and perinatal exposure to antibiotics can result in an increased risk of childhood asthma (139). Findings from the Copenhagen Prospective Study on Asthma in Childhood identified an increased risk of asthma with maternal use of antibiotics in the third trimester of pregnancy, and the Danish National Birth Cohort study showed an increased risk of asthma in children in response to maternal use of antibiotics irrespective of the pregnancy stage (140). Administration of intrapartum antibiotics, such as Group B Streptococcus antigen, can disturb the vaginal microbiome composition (141), leading to disruption of maternal microbiome inheritance in the offspring. As found in the Canadian Healthy Infant Longitudinal Development (CHILD) study, children of mothers who received intrapartum antibiotics exhibited microbial dysbiosis at three months of age, which was characterized by low levels of *Bacteroides* and high levels of *Enterococcus* and *Clostridium* (142). Administration of longer-term intrapartum antibiotics has been shown to increase the risk of atopic dermatitis in children by up to 28.9% at the age of two (143). Together, these studies identify the importance of both developmental and environmental factors in modulating the asthma risk profile in offspring and demonstrate additional mechanisms for the inheritance of non-genetically encoded factors that have a direct influence on the incidence of disease.

## Metabolomics and Proteomics Profiling in Asthmatics

In-depth metabolomic and proteomic analyses of samples from asthma patients have been assessed in a broad range of tissues including blood, urine, solid tissue biopsies, exhaled breath condensate (EBC), bronchoalveolar lavage fluid (BALF), sputum, and stool, showing distinct differential associations of proteins and metabolites with disease states (**Table 2**: Significant targets resulting from Metabolic and Proteomic studies in Asthmatic Patients). At a high-level view, the associations observed to correlate with asthma reflect those of general stress response mechanisms and involve both immune and inflammatory functions (144, 145), with many of the biomarkers identified, reflecting diverse inflammatory pathologies, which can be used to stratify asthma phenotypes and endotypes (146).

**Table 2:** Significant Targets Resulting from Metabolomic and Proteomic Studies of Samples Derived from Asthmatic Patients.

Sample	Type	Significant Targets	year	Reference (DOI)
Blood	Metabolomics	PC.ae.C42:1 and PC.ae.C42:5	2013	doi.org/10.1111/all.12110
		taurine, nicotinamide, AMP, and arachidonate in asthmatics, 1-steraroyylglycerol, degydroisoandrosterone sulfate, androsterone sulfate, valine, isoleucine, and ornithine	2015	doi.org/10.4049/jimmunol.1500736
		DHEA-S, cortisone, ProHyp, pipecolate, N-palmitoyltaurine, cortisol, S1P, N-palmitoltaurine, 22-hydroxycholesterol, xanthine, ceramides, sphingomyelins, eicosanoids, and fatty acid	2017	DOI: 10.1183/13993003.01740-2016
Plasma	Metabolomics	monoHETE0863, and sphingosine-1-phosphate, arachidonic acid, PGE2 and S1P	2015	doi.org/10.1002/iid3.61
		L-Arginine, B-Alanine,D,L-B-Aminoisobutyric Acid, Taurine,Ƴ-Amino-N-Butyric Acid, L-Tryptophan, L-Valine, L-Histidine, Hydroxy-L-Proline	2020	doi.org/10.3390/ijerph17134758
		Histidine, 1-methylnicotinamide, trimethylamine N-oxide (TMAO)	2020	doi.org/10.3390/jcm9030887
		SM 34:2, SM 38:1, SM 40:01:00	2021	doi.org/10.1002/JLB.3MA1120-719R
		Phosphatidylethanolamine (PE) (18:1p/22:6), Phosphatidylinositol (PI) (16:0/20:4), TG (20:0/18:1), PE (38:1), sphingomyelin (SM) (17:0/18:1/18:1), phosphatidylglycerol (PG) (44:0), PE(d18:1/18:1), triglyceride (TG)(16:0/16:0/18:1), ceramide (Cer) (d16:0/27:2), lysophosphatidylcholine (LPC) (22:4)	2021	doi.org/10.1016/j.bbalip.2020.158853
Serum	Metabolomics	Formate, methanol, acetate, choline, O-phosphocholine, arginine, and glucose	2013	doi.org/10.1111/cea.12089
		4-dihydroxybenzoic acid, 5-aminovaleric acid, ascorbate, dehydroascorbic acid, inosine, phenylalanine, and succinic acid (succinate), b-glycerophosphoric acid, maleamate, maleic acid, monoolein, ribose, and trans-4-hydroxy-L-proline	2015	DOI: 10.1038/aps.2015.102
		Ursodeoxycholic acid,Palmitic acid, Lauric acid Deoxycholic acid, Isodeoxycholic acid,EPA	2017	doi.org/10.1016/j.aca.2017.08.009
		Monosaccharides, Glycerophosphocholine, LysoPC (18:1), Retinyl ester, PC(18:1/2:0), LysoPC(o-18:0),PS(18:0/22:5), Arachidonic acid,Cholesterol glucuronide, PE(18:3/14:0), Phytosphingosine,PC (16:0/18:1), Sphinganine, LysoPC(p-18:1), Retinols, PC(20:4/16:1)	2018	doi.org/10.1155/2018/2860521
		Hypoxanthine, L-Glutamine, Glycerophosphocholine, P-chlorophenylalanine, Succinate, Xanthine, Arachidonic Acid, Inosine, Theophylline, L-Pyroglutamic acid, Bilirubin, Palmitic acid, Indoxyl sulfate, L-Valine, L-Norleucine, L-Leucine, L-Phenylalanine	2019	DOI: 10.3967/bes2019.085
		2-palmitoylglycerol, cholesterol, serine, threonine, Lactic acid, 2-palmitoylglycerol, Ethanolamine, Glucose, Stearic acid, Linoleic acid, D-Mannose, Succinic acid	2020	DOI: 10.1186/s12931-020-01390-4
		Succinate N-(2-furoyl)glycine, Iminodiacetate (IDA)	2021	doi.org/10.1016/j.jaci.2020.12.639
EBC	Metabolomics	Leukotrienes	2009	doi.org/10.1016/j.jchromb.2009.01.036
		Adenosine	2009	doi.org/10.1152/ajplung.90512.2008
		Nonane, 2,2,4,6,6-pentamethylheptane, decane, 3,6-dimethyldecane, dodecane, tetradecane	2012	doi.org/10.1016/j.chroma.2012.07.023
		Retinoic acid, adenosine, and vitamin D	2012	doi.org/10.1111/all.12063
		1,4-dichlorobenzene, 4-isopropenyl-1methylcyclohexene, 2-octenal, octadecyne, 1-isopropyl-3methylbenzene, 1, 7-dimethylnaphtalene	2013	doi.org/10.4155/bio.13.184
		Saturated fatty acids, valine, adenosine, hippurate, alanine, formate, urocanic acid, proline, acetate, ethanol, methanol, isoleucine, propionate,	2014	DOI: 10.1021/pr5010407
		uracil, urocanic acid, succinate, SFA, Phe, hippurate, trimethylamine, 4OH-phenylacetate, Val, acetate, SFA, Pro, Tyr, Arg, trans-aconitate,succinate, Val, propionate, SFA, methanol, uracil, Pro, formate, isobutyrate, urocanic acid, adenosine, Hippurate, Ala, acetate, ethanol, methanol, and Ile	2014	doi.org/10.1021/pr5010407
		Alkanes, acetone, 2, 4-dimethylpentane, 2, 4-dimethylheptane, 2,2, 4-trimethylheptane, 1-methyl-4-(1methylethenyl) Cyclohexen, 2,3, 6-trimethyloctane, 2-undecenal, Biphenyl, 2-ethenylnaphtalene, 2,6, 10-trimethyldodecane, Octane, 2-methylpentane, 2,4-dimethylheptane, and 2-methylhexane	2014	doi.org/10.1371/journal.pone.0095668
		isopropanol and N,N, dimethylglycine and ammonia	2017	DOI: 10.1186/s12967-017-1365-7
		glyoxylate, dicarboxylate, pyruvate, glucose, butyrate, acetoin, formate, tyrosine, ethanol, ethylene glycol, methanol, n-valerate, acetate, SFA, propionate, n-valerate, acetoin, isovalerate, 1,2-propanediol, ethnol, acetone, propionate, acetate, lactate, and SFA	2017	doi.org/10.1016/j.jaci.2016.08.038
		lysine, eicosanoids, phospholipids	2020	doi: 10.1088/1752-7163/ab9220
		9-amino-nonanoic acid, 12-amino-dodecanoic acid, lactone of PGF-MUM, N-linoleoyl taurine, 17-phenox trinor PGF2α ethyl amide, lysoPC [18:2(9Z,12Z)]	2020	doi.org/10.3390/metabo10100390
Sputum	Proteomics	SERPINA1S100A9, S100A8, SMR3B, and SCGB1A1	2011	doi.org/10.1016/j.jaci.2011.07.053
		S100A9	2013	DOI: 10.1016/j.anai.2013.06.028
	Metabolomics	Glycerol 1-stearate_1, 1-Hexadecanoyl-snglycerol_1, Cytidine His-Pro, Thr-Phe_1, 20,30-cyclic phosphate, Arg-Phe_1, Adenine_1, Phe-Tyr_1, Phe-Gln_1, 1-Hexadecanoyl-2-(9Zoctadecenoyl)-sn- Tyr-Ala_2, Phe-Ser_1, glycero-3-phospho-(10rac-glycerol), Urocanic acid 1-Octadecanoyl-2-(9Zoctadecenoyl)-snglycero-3phosphoserine	2017	PMCID: PMC6965799
BALF	Proteomics	CLC, MBP, EDN, ECP, CRISP-3, and MMP-9	2005	doi.org/10.1074/mcp.M500041-MCP200
		IL-4 and gelsolin	2005	doi.org/10.1164/rccm.200409-1185OC
		galectin-3	2012	doi.org/10.1016/j.bbagen.2011.12.016
	Metabolomics	lysophosphatidylcholine (LPC), phosphatidylcholine (PC), phosphatidylglycerol (PG), phosphatidylserine (PS), sphingomyelin (SM), triglyceride (TG)	2014	doi.org/10.1021/pr5002059
Urine	Metabolomics	2-oxaloglutarate, succinate, fuma- rate, 3-hydroxy3-methylglutarate, threonine, and cis-aconitate and trans-aconitate	2011	DOI 10.1016/j.jaci.2010.12.1077
		Urocanic acid and methylimidazoleacetic acid	2012	doi.org/10.1002/bmc.1631
		Threonine, lactate, alanine, carnitine, acetylcarnitine, trimethylamine-N-oxide, acetate, citrate, malonate, hippurate, dimethylglycine, and phenylacetylglutamine	2014	DOI 10.1016/j.jaci.2013.11.004
		Glutamine, succinate, uracil, pantothenate, Arginine, dimethylamine, 3-Hydroxyisovalerate, betaine, choline, glucose, 1-methylnicotinamide	2015	doi.org/10.1016/j.jaci.2015.05.022
		4-(4-deoxy-α-d-gluc-4enuronosyl)-d-galacturonate, Oxoadipic acid(-)-epinephrine, l-tyrosine, Glutaric acid,4-hydroxynonenal, 3-hydroxyhippuric acid Benzoic, 3-hydroxy-sebacic acid, Dihydroferulic acid 4-sulfate, 3-methyluridine, Steroid O-sulfate, 5hydroxy-l-tryptophan, 3-Indolelactic acid, 3-indoleacetic acid, N2-acetyl-ornithine, Tiglylglycine, Indole, Cytosine, N-acetylputrescine, Indole-3-acetamide, 6-methyladenine, 5-methylcytosine, N-acryloylglycine, Hydroxyphenyllactic acid	2018	doi.org/10.1111/pai.12879
		Guanidoacetic acid, 1-methylnicotinamide, allantoin	2018	doi.org/10.1111/pai.12909
		bile acid taurochenodeoxycholate 3-sulfate, fatty acid 3-hydroxytetradecanedioic acid, glucoronidated steroid	2018	doi.org/10.3390/metabo9090185
		Aspartic acid, Stearic acid, Xanthosine, Heptadecanoic acid, Hypoxanthine, Uric acid, D-threitol, N-acetylgalactosamine	2019	doi.org/10.1111/resp.13479
		L-allothreonine 1, stearic acid, succinic acid, Valine, uric acid, methionine 1, 2-hydroxybutanoic acid, azelaic acid, 3,4-dihydroxycinnamic acid, purine riboside, gentiobiose 2, tyramine, leucine, D-altrose 1, malonic acid 1, cysteine, erythrose 1, lactamide 1, D-erythrosphingosine 1, citraconic acid 4	2020	DOI: 10.2147/JAA.S281198

EBC is a very practical non-invasive method to collect samples, although it is still currently limited in the range of metabolites that can be detected. EBC methodology has been successfully applied, whereby differential metabolic profiles between healthy subjects and the severity of asthma subtypes could be readily distinguished (147–149). In comparison, proteomic analyses of asthmatic EBC and sputum show more fine-grained results, indicating alterations in the inflammatory state including levels of α1 antitrypsin, α2 macroglobulin, SERPINs, S100-family proteins, apolipoproteins, complement proteins, and Th2-related cytokines (150, 151). Whereas BALF and induced sputum samples have the potential to reflect the local pathophysiological state in a more direct and accurate manner, they are highly invasive and difficult to obtain. Interestingly, an analysis of a lipid repertoire present following metabolomic analysis of asthma patients noted that increases observed in asthmatic BALF samples were similar to those observed in the sputum and urine samples and mainly involved those lipids from glycerophospholipid and fatty acid metabolism associated pathways (152–154). Plasma metabolomics and proteomics patterns have also been used to indicate the presence of multiple and different asthma disease states within the broader population of asthmatic patients. Here, molecules involved in inflammation were found to include chaperone proteins, interferon response elements and leukocyte migration factors which were identified alongside those of allergy associated Th2 cytokines and were found to be differentially expressed between sub-phenotypes (155, 156). Additionally, studies focused on pediatric asthmatic populations have identified tryptophan, tyrosine, and biliary acids metabolism associated metabolites in the urine and plasma as markers that were predictive of the development and severity progression of the disease (157).

As such both protein and metabolite biomarkers are beginning to provide powerful new means to develop personalized diagnoses, treatments, and prognoses for asthma patients. Additionally, the use of these biomarkers should prove to be a highly useful tool for the accurate stratification of asthma endotypes/phenotypes when recruiting patients for GWAS. However, a better understanding of the impact and crosstalk between discovered biomarkers with the immune system is still needed not only to better define the variety of phenotype and endotypes associated with asthma, but also to evaluate the risk factors associated with developing asthma. Recently, the microbial metabolome has emerged as a new player that also requires consideration for both asthma predisposition and severity (158), with data indicating that microbial metabolites such as the short chain fatty acids butyrate (159) and propionate (160) are highly important for inducing the T regulatory subsets associated with homeostatic maintenance. Whereas other metabolites including 12,13-diHOME, may be responsible for induction of disease through induction of Th2 differentiation (2, 161) and recruitment of type 2 inflammatory cells including ILC2’s. Hence, future studies to form an in-depth understanding of the proteomic and metabolomic profiles present during pregnancy, stemming from both maternal and microbial sources, will be required to formally investigate how these factors may contribute to a pro-atopic state.

## Current Application of Multiomics Analysis to Asthma Studies

While relatively few truly multiomics analyses of asthma have been generated to date, the breadth of data acquired so far in terms of epigenomic, genomic, metabolomic, microbiomic, proteomic and transcriptomic is quite staggering. This holds great promise for both application and incorporation of existing datasets for metanalytical studies and for use in complementing *de novo* studies. A recent methodology for utilization of omics data generated from multiple sources describes a promising pipeline for generating multiomics analyses from data collected in multiple studies, known as a Genome Wide Cross-Trait analysis Study (162). Here, multiple analyses including genetic correlation, cross-trait metanalysis, Mendelian randomization, PRS, and GWAS functional analysis are combined in an integrated manner to study genetic and functional effects on disease outcomes and associations. The application of this approach to data collected in the UK biobank, led to the identification of 38 genome-wide significant loci of which 7 were novel to the study and further analysis revealed that the shared loci were specifically enriched in immune/inflammatory systems and epithelial cells (163).

A similar strategy taken in Han et al. (19), began with the metanalysis of multiple GWAS datasets, analyzed enrichment of asthma associated loci at epigenetic markers, and incorporated EQTL data from GTEx Project and eQTLGen Consortium databases. This enabled the prioritization of loci for downstream verification, leading to the identification and functional validation of *CD52* as a candidate causal gene for asthma and demonstrating the utility of integrating multiple omics datasets for target discovery. Additionally, multiomics approaches have been leveraged in order to better define asthmatic endotypes and to examine their longitudinal relation with asthma risk (164). Here, retroviral typing microbiome, cytokine, and metabolome data collected upon admission to hospital with bronchiolitis at <12 months of age were used in order to generate a model that identified biologically relevant bronchiolitis endotypes in infants who were at increased risk of developing asthma at 3 years of age.

Studies such as these highlight the importance and potential utility of applying multiomics based approaches to the study of asthma. However, significant difficulties still exist in comparing data generated on differing technologies due to both data heterogeneity and inherent biases in the systems used to generate these datasets. With the continued development of new methods and tools for bioinformatics analysis, including machine learning and deep learning techniques, these will undoubtedly help to ease the bottlenecks associated with the integration of high dimensional data generated from multiple sources in multiple modalities (165, 166).

## Conclusion

The application of large-scale genome-wide association studies (GWAS) in asthma and atopic diseases over the last decade has led to the determination of a large number of gene variants linked to these conditions. Despite the power of genetic studies, the variants identified generally have small odds ratios and have only been able to partially explain the predicted heritability associated with the transfer of disease to the next generation. The rapid rise in the rates of these conditions over the past 50 years indicates that alterations in gene-environment and gene-developmental factors play a substantial role in the onset of disease, with multiple studies indicating that the identification of modifiable risk factors can exert a significant impact on the incidence of asthma. Furthermore, the observation that maternal incidence of asthma is significantly more predictive of childhood disease can now in part be explained by studies demonstrating that the transfer of immune factors during pre- and post-natal conditioning play a significant role in setting disease susceptibility thresholds. As the immune system first encounters the external environment and adapts to the subsequent colonization of the body by the microbiome, maternal factors play a key role during this time of life that is uniquely sensitive in terms of establishing a set point for a pro-atopic state. Future studies aimed at the integration of genomic risk factors *via* PRS in combination with multiomics analysis of cellular, epigenetic, and microbiome-influenced conditioning will allow the development of more accurate models, for both the prediction of disease, as well as for the application of targeted approaches aimed at reducing the disease prevalence.

## Author Contributions

All authors listed have made a substantial, direct, and intellectual contribution to the work, and approved it for publication.

## Conflict of Interest

The authors declare that the research was conducted in the absence of any commercial or financial relationships that could be construed as a potential conflict of interest.

## Publisher’s Note

All claims expressed in this article are solely those of the authors and do not necessarily represent those of their affiliated organizations, or those of the publisher, the editors and the reviewers. Any product that may be evaluated in this article, or claim that may be made by its manufacturer, is not guaranteed or endorsed by the publisher.
